# microRNA-140 Regulates PDGFRα and Is Involved in Adipocyte Differentiation

**DOI:** 10.3389/fmolb.2022.907148

**Published:** 2022-06-27

**Authors:** Yi Yan, Jiahui Yuan, Xiaomao Luo, Xiuju Yu, Jiayin Lu, Wei Hou, Xiaoyan He, Liping Zhang, Jing Cao, Haidong Wang

**Affiliations:** ^1^ College of Veterinary Medicine, Shanxi Agricultural University, Jinzhong, China; ^2^ Department of Medicine, Nephrology Division, Baylor College of Medicine, Houston, GA, United States; ^3^ Department of Animal Husbandry and Veterinary Medicine, Beijing Vocational College of Agriculture, Beijing, China

**Keywords:** miR-140-5p, adipocyte, 3T3-L1 cells, differentiation, adipogenesis

## Abstract

In recent years, the studies of the role of microRNAs in adipogenesis and adipocyte development and the corresponding molecular mechanisms have received great attention. In this work, we investigated the function of miR-140 in the process of adipogenesis and the molecular pathways involved, and we found that adipogenic treatment promoted the miR-140-5p RNA level in preadipocytes. Over-expression of miR-140-5p in preadipocytes accelerated lipogenesis along with adipogenic differentiation by transcriptional modulation of adipogenesis-linked genes. Meanwhile, silencing endogenous miR-140-5p dampened adipogenesis. Platelet-derived growth factor receptor alpha (PDGFRα) was shown to be a miR-140-5p target gene. miR-140-5p over-expression in preadipocyte 3T3-L1 diminished PDGFRα expression, but silencing of miR-140-5p augmented it. In addition, over-expression of PDGFRα suppressed adipogenic differentiation and lipogenesis, while its knockdown enhanced these biological processes of preadipocyte 3T3-L1. Altogether, our current findings reveal that miR-140-5p induces lipogenesis and adipogenic differentiation in 3T3-L1 cells by targeting PDGFRα, therefore regulating adipogenesis. Our research provides molecular targets and a theoretical basis for the treatment of obesity-related metabolic diseases.

## Introduction

As an important tissue of the body, adipose tissue participates in controlling the body’s overall energy balance, and the dysfunction of adipose tissue may induce cardiovascular diseases and type 2 diabetes (Shamsi et al., 2021). Adipocytes, as the key element of adipose tissue, is of great importance for maintaining systemic metabolism balance (Rajala and Scherer, 2003; Park et al., 2011). Adipocytes have some special functions. First, they can release adipokines to perform specific endocrine functions. Secondly, they can also store excessive energy as triglycerides in adipocytes to maintain systemic energy balance (Cohen and Spiegelman, 2016). Adipocyte hypertrophy and/or adipocyte hyperplasia tend to result in adipose tissue accumulation (Wang et al., 2018). Previous research has shown that adipogenic differentiation is the process responsible for adipocyte hyperplasia (Lee et al., 2017).

The exploration of the signaling pathway of adipocyte differentiation is critical not only for understanding adipogenesis, but also for developing new therapeutic targets for metabolic diseases, including diabetes and obesity (Song et al., 2017). Previous studies have identified nuclear proteins, peroxisome proliferator-activated receptors (PPARs), and CCAAT/enhancer-binding proteins (C/EBPs) as key regulators of adipocyte formation and adipocyte differentiation, and these two transcription factors jointly regulate the expression of the lipid metabolism-related genes (He et al., 2008; Villanueva et al., 2011).

Recent studies have suggested that microRNAs (miRNAs) seem to play major roles in adipocyte differentiation (Shi et al., 2016; Vienberg et al., 2017). MiRNAs can affect adipogenesis-related signaling pathways, target transcription factors, block the clonal expansion stage of mitosis, and accelerate or delay adipocyte differentiation during adipogenesis, thereby regulating adipocyte formation (Chen et al., 2013; Wosczyna et al., 2021). The miR-140 is produced by the miR-140 gene. A previous study has reported that miR-140 is extensively, highly, and selectively expressed in chondrocytes (Papaioannou et al., 2015). Recent investigations have documented that miR-140 is involved in tumor growth, metastasis, and pulmonary arterial hypertension (Yang et al., 2013; Rothman et al., 2016). However, the effects of miR-140 on adipogenesis and adipocyte development, especially on adipocyte differentiation regulation, remain largely unclear. miR-140 has recently been found to regulate the lipid accumulation and atherosclerosis and to participate in the differentiation of C3H10T1/2 cells (Liu et al., 2013; Zhao et al., 2021). Furthermore, C/EBP enhances miR-140-5p expression by activating its promoter transcript, and it is related to adipocyte differentiation (Zhang et al., 2015). The aforementioned studies have shown that miR-140 plays a crucial part in adipocyte differentiation.

Platelet-derived growth factor (PDGF) activates various cell processes, for instance, angiogenesis, cell proliferation along with differentiation, and cell survival by binding to α and β tyrosine kinase receptors of PDGFRα or PDGFRβ (Heldin, 2013). Upon binding of PDGF to PDGFRα or β, the α and β subunits dimerize, thus activating the intrinsic tyrosine kinase activity of these receptors eventually activating a series of PDGFRα or PDGFRβ downstream signaling cascades (Kikuchi and Monga, 2015). According to previous reports, PDGF-related signaling cascades are involved in the onset of fibrosis, cancers, and atherosclerosis (Stock et al., 2007; Zhang et al., 2007; Zhang et al., 2009). PDGFRα regulates cellular proliferation, differentiation, and development of multiple tissues from embryogenesis to adulthood (Andrae et al., 2008).

Recently, researchers found that PDGFRα has been implicated in the adipocyte lineage as it is expressed in adipogenic stromal cells and adipocyte stem cells (ASC) (Berry et al., 2014; Burl et al., 2018). Previous studies have shown that PPARγ activation mediated the inhibition of PDGFRα expression in vascular smooth muscle cells (VSMCs) by inhibiting C/EBP. Nevertheless, the role of PDGFRα in adipocyte function and adipocyte differentiation is largely unknown. Recently, it was shown that miR-140-5p over-expression suppressed the expression of PDGFRα in cultured mouse palate cells (Li et al., 2020). Furthermore, reports have shown that GO enrichment analysis was conducted for the target genes of miR‐140‐5p predicted by at least three databases and found that PDGFRα was positively predicted as downstream targets of miR‐140‐5p (Li et al., 2017a). Although the aforementioned studies indicated that PDGFRα may be a target gene of miR-140, no further *in vivo* or *in vitro* studies were carried out. Especially, their molecular function in adipocyte differentiation remains unknown.

Herein, our data illustrated that the expression of miR-140-5p was elevated during adipocyte differentiation. The involvement of miR-140-5p in modulating lipid droplet generation and the expression of adipogenesis-linked genes was also elucidated. Furthermore, we revealed the mechanism by which platelet-derived growth factor receptor alpha (PDGFRα) modulates adipocyte differentiation. Our data illustrated that the cross talk of miR-140-5p and PDGFRα 3′-untranslated regions (3′UTR) induced post-transcriptional silencing, thus resulting in the stable expression of adipogenesis-linked genes and the maintenance of 3T3-L1 cell properties during adipocyte differentiation.

## Results

### Expression of miR-140-5p is Modulated During Adipocyte Differentiation

**FIGURE 1:**
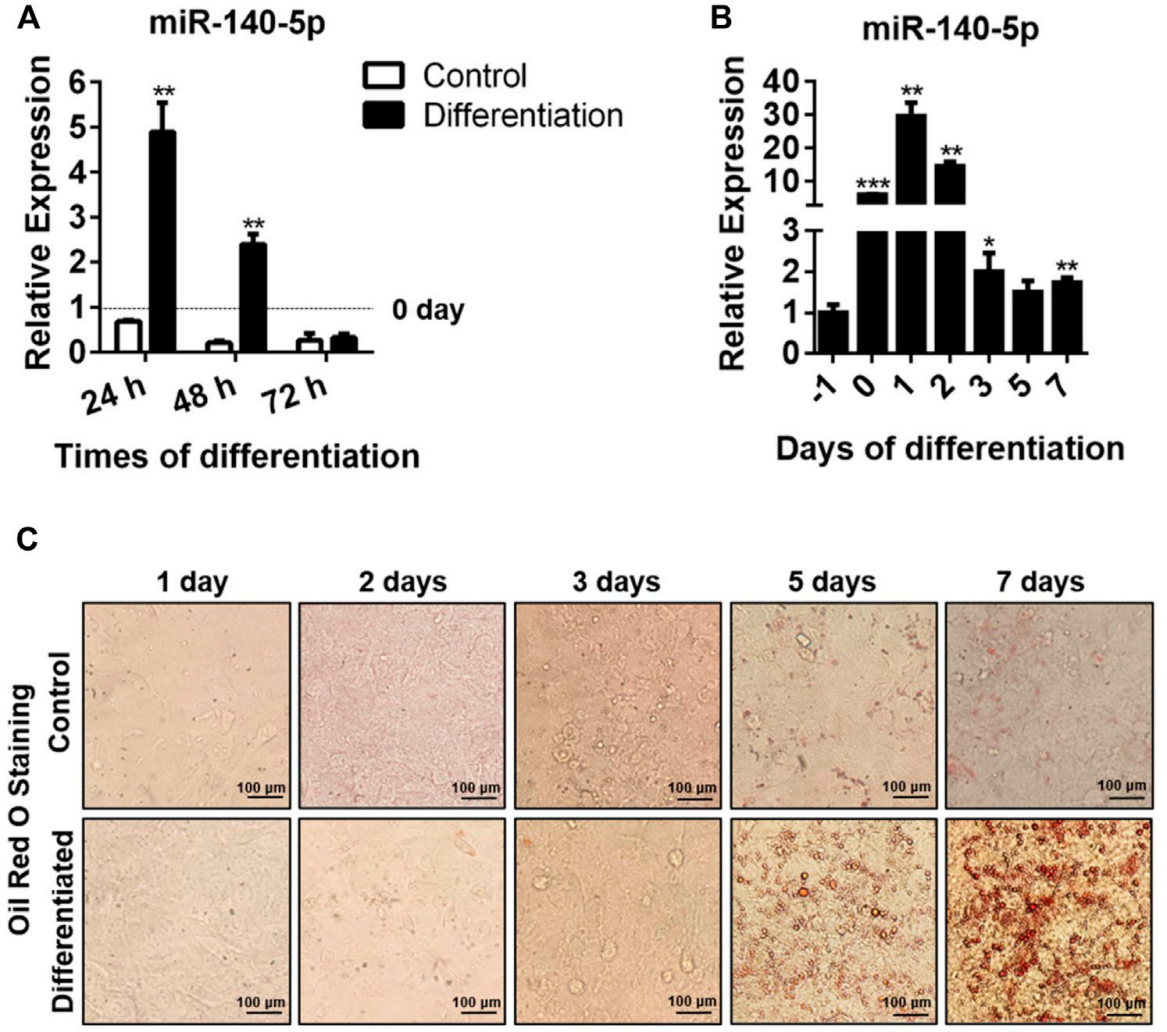
miR-140-5p expression during adipocyte differentiation. **(A)** Expression of miR-140-5p at 24, 48, and 72 h during differentiation of preadipocytes treated with differentiation cocktail or DMI based on qRT-PCR. miR-140-5p expression in this study was normalized with U6 as the internal control. **(B)** miR-140-5p expression at diverse time points (1 day before differentiation, 0, 1, 2, 3, 5, and 7 days) during differentiation of preadipocytes. 3T3-L1 cells were inoculated with differentiation cocktail or DMI based on qRT-PCR. **(C)** Oil red O staining of preadipocytes at diverse time points (1, 2, 3, 5, and 7 days) during differentiation (100-fold magnification). Data are given as mean ± SD (n = 3, with three replicates in one independent experiment). Remarkable difference is given at the levels of *p < 0.05, **p < 0.01, and ***p < 0.001 via two-tailed Student’s t test.

We hypothesized that miR-140-5p could act as an element modulating the adipocyte differentiation process and that the expression of miR-140-5p varied with differentiation time. Consistent with our expectation, we found that miR-140-5p mRNA level reached its highest at 24 h and declined afterward (Figure 1A,B). In addition, we found that the formation of lipid droplets was gradually elevated with the extension of differentiation time and reached the maximum after 7 days of induction by oil red O staining (Figure 1C).

### MiR-140-5p Is Identified as Adipogenic Factor

**FIGURE 2:**
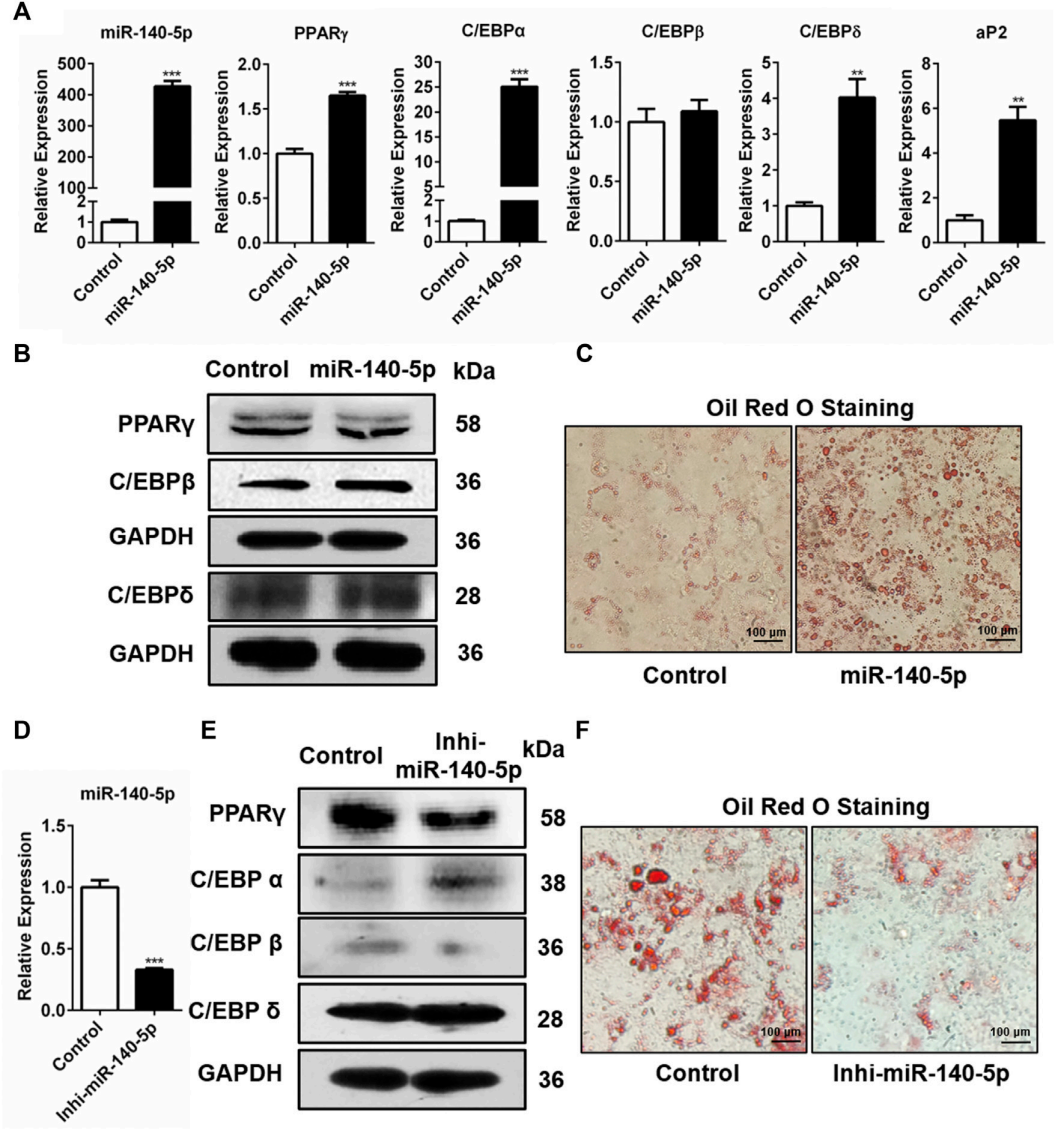
Adipogenesis regulation via miR-140-5p as the adipogenic factor. **(A)** Expression levels of miR-140-5p and adipogenic genes after transient transfection with negative control mimics (Control) or miR-140-5p mimics in 3T3-L1 cells based on qRT-PCR analysis. **(B)** Western blot assessment of expression level of adipogenesis-linked genes after 3T3-L1 cells were transfected with miR-140-5p mimics, as well as control mimics. **(C)** Oil red O staining of 3T3-L1 cells in cell transfects of miR-140-5p mimics and control mimics (100-fold magnification). **(D)** Expression levels of miR-140-5p in 3T3-L1 preadipocytes transfected with miR-140-5p inhibitors (Inhi-miR-140-5p) and negative control (Control) based on qRT-PCR analysis. **(E)** Adipogenesis-linked gene protein content in 3T3-L1 cell transfects of miR-140-5p inhibitor or control inhibitor. **(F)** Oil red O staining of preadipocytes—3T3-L1 in the miR-140-5p inhibitor transfection group (Inhi-miR-140-5p) and control group (100-fold magnification).

Further, we studied the influence of miR-140-5p on adipogenic transcription factors in 3T3-L1 cells. miR-140-5p was overexpressed by transfecting miR-140-5p mimics and negative control (NC) into 3T3-L1 cells. Our results showed that the transcription contents of C/EBPδ, PPARγ, C/EBPα, and adipocyte fatty acid binding protein (aP2) drastically increased in miR-140-5p mimic transiently transfected cells (Figure 2A). But there was no significant change in the expression level of C/EBPβ after over-expression of miR-140-5p (Figure 2A). Consistently, the protein contents of PPARγ, C/EBPβ, and C/EBPδ were significantly higher after transfecting miR-140-5p (Figure 2B, Supplementary Figure S1A). Oil red O staining results illustrated that the transfection of miR-140-5p strongly induced the lipid droplet formation in preadipocytes (Figure 2C). These findings imply that miR-140-5p accelerated lipid droplet generation via expediting adipogenesis in 3T3-L1 preadipocytes.

Moreover, miR-140-5p inhibitor was inserted into 3T3-L1 preadipocytes via transfection. After the miR-140-5p inhibitor was transfected into 3T3-L1 cells, the expression of miR-140-5p was significantly reduced (Figure 2D). Western blotting data revealed that the knockdown of miR-140-5p resulted in reduced PPARγ and C/EBPβ protein expressions compared with the NC group (Figure 2E, Supplementary Figure S1C). However, qRT-PCR data illustrated that silencing of miR-140-5p induced no statistically significant difference in the expression of adipogenesis-linked genes (Supplementary Figure S1B). Oil red O staining analysis revealed that the silencing of miR-140-5p reduced lipid production and accumulation in 3T3-L1 cells (Figure 2F). Overall, our data illustrate that miR-140-5p acts as an important regulator for adipocyte differentiation.

### PDGFRα Is Identified as a Direct Target of miR-140-5p

**FIGURE 3:**
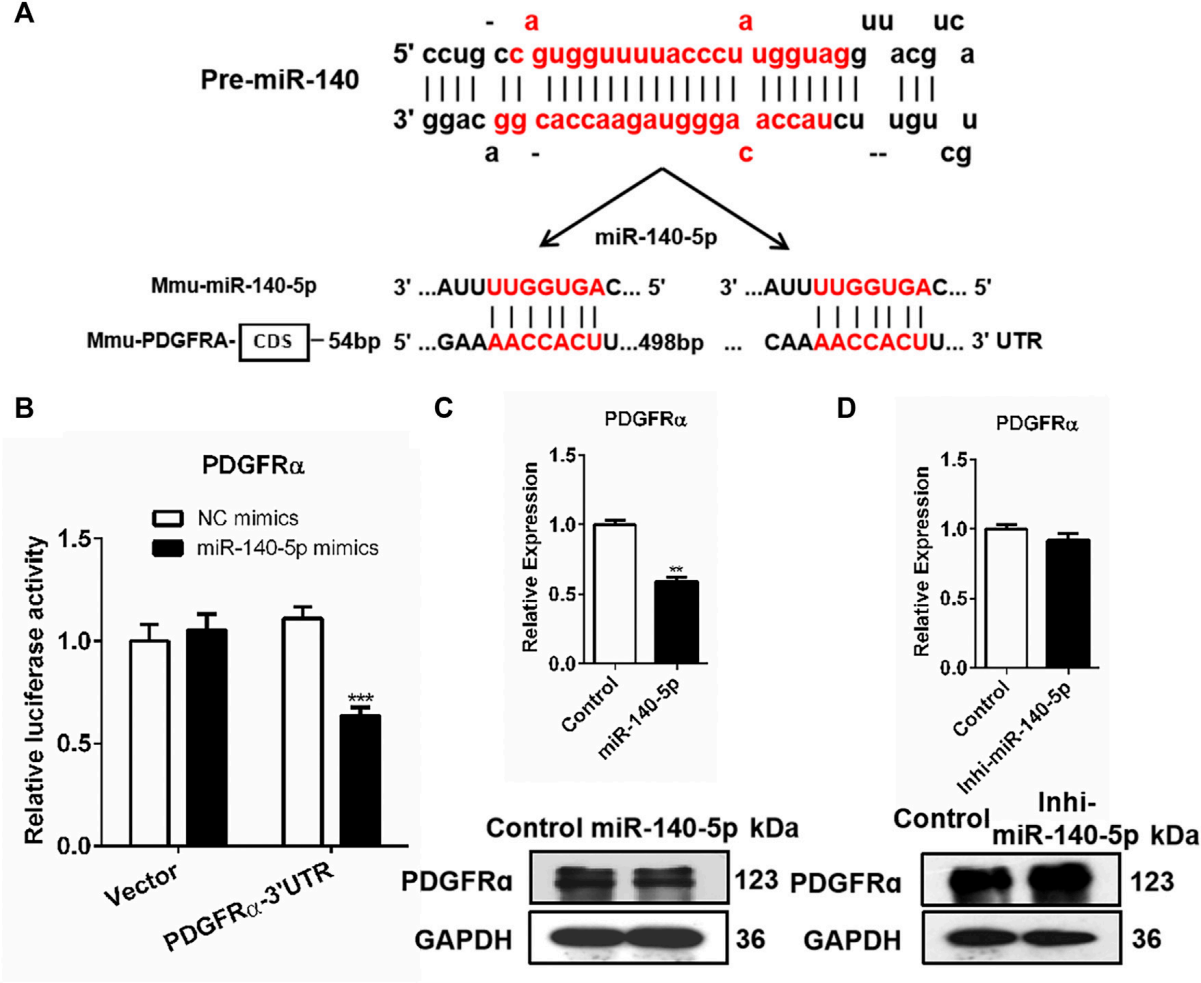
PDGFRα as a direct target of miR-140-5p. **(A)** Docking site sequence of miR-140-5p to 3′UTR of PDGFRα gene according to the TargetScan database. **(B)** Relative luciferase enzyme activity of PDGFRα-3′UTR vector and control vector after co-transfection with miR-140-5p mimics (miR-140-5p) or negative control mimics (Control) in HEK293T cells. **(C)** PDGFRα mRNA and protein levels after transient transfection of 3T3-L1 cells with miR-140-5p mimics or control based on qRT-PCR and Western blot. **(D)** PDGFRα expression levels after transient transfection of 3T3-L1 cells with miR-140-5p inhibitors (Inhi-miR-140-5p) or control based on qRT-PCR and Western blot.

We screened the putative mRNA from the candidate target genes contributing to adipocyte differentiation downstream miR-140-5p against the miRbase and TargetScan databases. Previous research has reported that the stimulation of the PDGFRα signaling pathway possibly restricts the differentiation of adipocyte precursor cells into adipocytes (Haider and Larose, 2019). Based on this, we speculated that miR-140-5p promoted adipogenesis via PDGFRα. Our analysis indicated that the PDGFRα gene’s 3′UTR contained miR-140-5p target sequences (Figure 3A). To determine whether PDGFRα was a direct target of miR-140-5p, a luciferase reporter vector was constructed by putting PDGFRα 3′UTR behind the luciferase gene. We co-transfected the vector or PDGFRα 3′UTR with NC mimics or miR-140-5p mimics into 293T cells. In comparison to the control group, the PDGFRα 3′UTR vector luciferase enzyme activity was repressed after co-transfection with miR-140-5p. Furthermore, co-transfection with either the NC or miR-140-5p mimics exhibited no change in the luciferase enzyme activity of the vector (Figure 3B).

Consistent with miRNAs’ post-transcriptional mechanism, PDGFRα displayed a significant reduction at both the mRNA level and protein level after miR-140-5p mimics’ transfection (Figure 3C, Supplementary Figure S2A). The PDGFRα mRNA level was evaluated by qRT-PCR after miR-140-5p inhibitors or NC inhibitor was transfected into 3T3-L1 cells. Transfection with miR-140-5p inhibitor resulted in no remarkable variation in PDGFRα mRNA levels, but there was a significant elevation in the protein level (Figure 3D, Supplementary Figure S2B). These findings indicate that PDGFRα is miR-140-5p’s direct target.

### Over-Expression of PDGFRα Suppresses Expression of the Adipogenesis-Linked Genes and Impairs Lipid Droplet Synthesis

**FIGURE 4:**
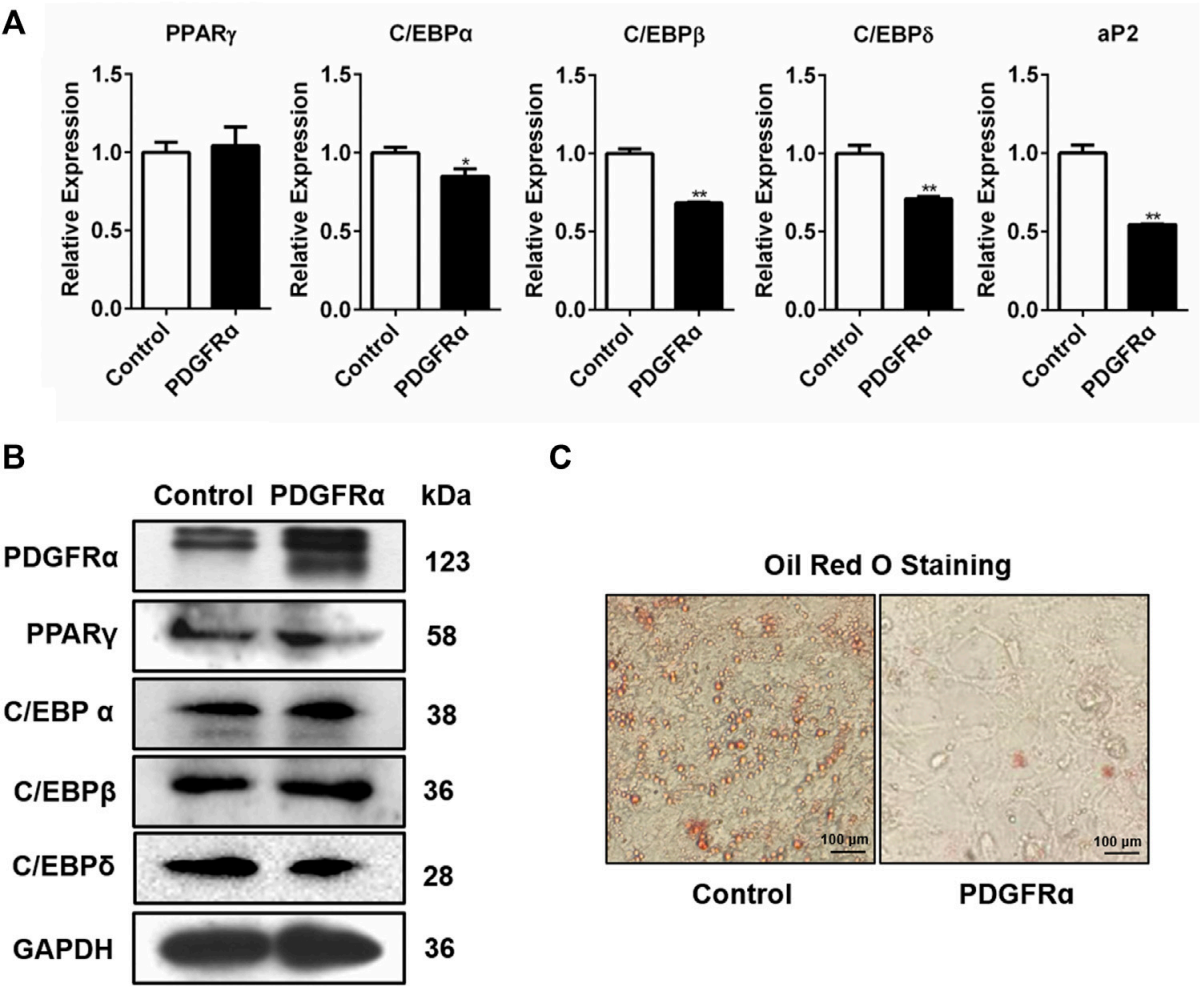
Influence of PDGFRα on adipogenesis-linked gene expression and lipid droplet synthesis in 3T3-L1 cells. **(A)** qRT-PCR analysis of the expression of adipogenesis-associated genes after transfection with pEGFP-N1-PDGFRα (PDGFRα) or control in 3T3-L1 cells. **(B)** Western blotting analysis of the expression of adipogenesis-associated transcription factors after transfection with pEGFP-N1-PDGFRα (PDGFRα) or control in 3T3-L1 cells. **(C)** Illustrative images of oil red O after transient transfection with pEGFP-N1-PDGFRα (PDGFRα) or control (100-fold magnification) in 3T3-L1 cells.

We also assessed how PDGFRα influences the expression of adipogenic transcription factors. We transfected 3T3-L1 cells with pEGFP-N1-PDGFRα to enhance PDGFRα function and found that aP2, C/EBPα, PPARγ, C/EBPβ, and C/EBPδ expressions significantly decreased in pEGFP-N1-PDGFRα–transfected 3T3-L1 cells (Figure 4A), indicating that PDGFRα dampened the expression of adipogenic transcription factors during adipocyte differentiation. Western blot assay data of the protein samples confirmed the reliability and validity of qRT-PCR results, illustrating that over-expression of PDGFRα resulted in a decrease in C/EBPα, PPARγ, C/EBPβ, along with C/EBPδ protein content (Figure 4B, Supplementary Figure S3). Oil red O staining analysis demonstrated that over-expression of PDGFRα effectively inhibited lipid production and accumulation (Figure 4C). Overall, the above results indicate that PDGFRα negatively influences adipogenesis-related gene expression and lipid droplet synthesis.

### PDGFRα Knockdown by Specific siRNA Increases Adipogenesis-Related Gene Expression and Intracellular Lipid Accumulation

**FIGURE 5:**
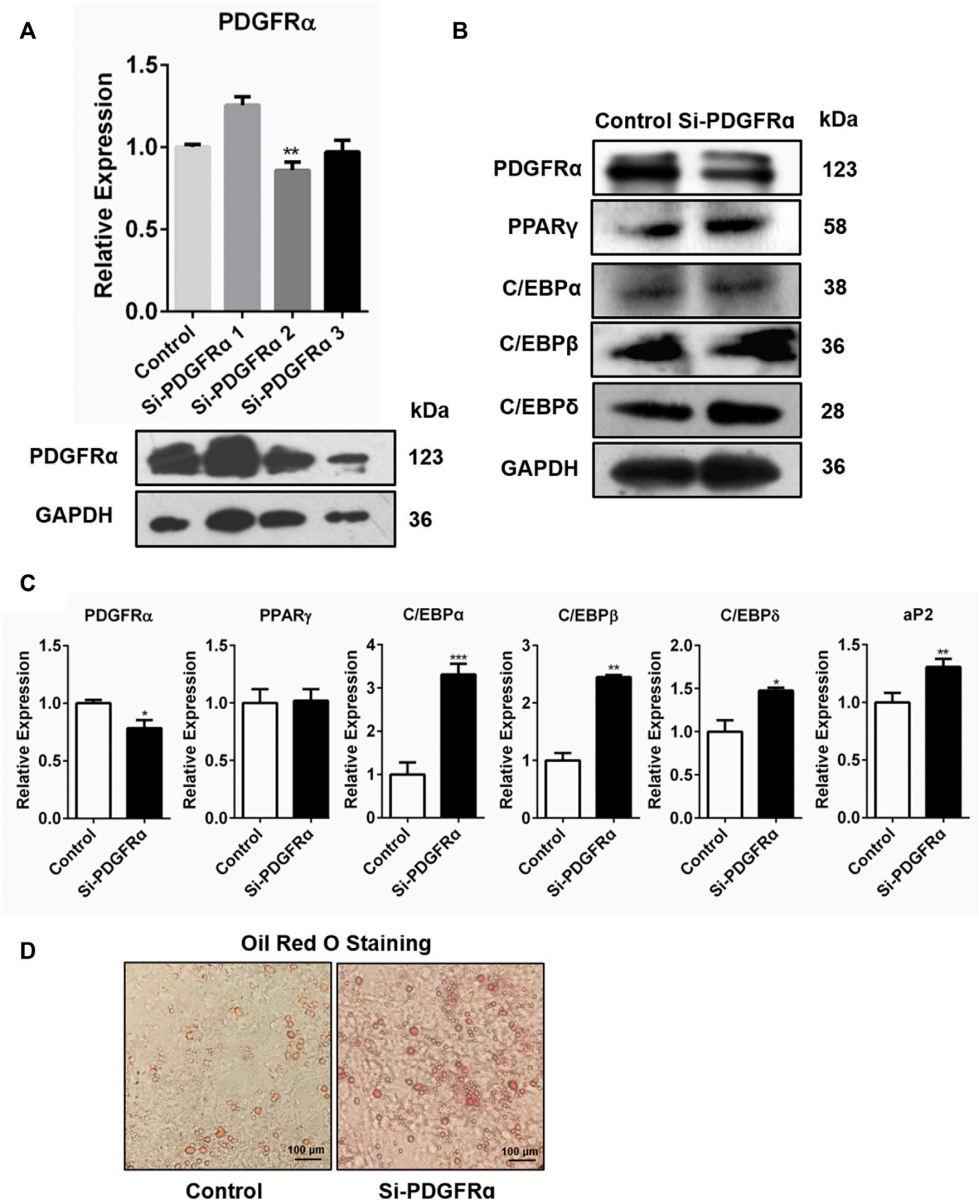
Increase in adipocyte differentiation-related gene expression and enhancement in lipid droplet synthesis by PDGFRα knockdown. **(A)** qRT-PCR along with Western blot assays of PDGFRα expression after transfection of 3T3-L1 cells with PDGFRα siRNA 1(Si-PDGFRα1), PDGFRα siRNA 2(Si-PDGFRα2), and PDGFRα siRNA 3(Si-PDGFRα3), respectively. **(B)** Western blot assessment of the expression of adipocyte differentiation-related transcription factors after being transfected with PDGFRα siRNA (Si-PDGFRα) or control in 3T3-L1 cells. **(C)** RT-PCR analysis of mRNA expression of adipocyte differentiation-related genes after transfection with PDGFRα siRNA (Si-PDGFRα) or control in 3T3-L1 cells. **(D)** Illustrative images of oil red O after transient transfection with PDGFRα siRNA (Si-PDGFRα) or control in 3T3-L1 cells (100-fold magnification).

To determine whether PDGFRα was engaged in adipogenesis, PDGFRα-specific siRNA was used to knock down PDGFRα in 3T3-L1 cells (Figure 5A, Supplementary Figure S4A). qRT-PCR along with Western blot assay data illustrated that transfection of 3T3-L1 cells, respectively, with Si-PDGFRα 1, 2, and 3 reduced PDGFRα function (Figure 5A) and that the expressions of adipogenesis-associated transcription factors were dramatically increased (Figures 5B,C, Supplementary Figure S4B). In addition, oil red O staining results illustrated that the knockdown of PDGFRα dramatically induced triacylglycerol accumulation in 3T3-L1 cells (Figure 5D). The aforementioned results exhibited that PDGFRα knockdown induced adipogenesis-linked gene expression and promoted intracellular lipid accumulation.

Overall, our data illustrated that miR-140-5p activated the adipogenesis-associated transcription factors and enhanced intracellular triacylglycerol accumulation, thus promoting adipogenesis by targeting PDGFRα.

## Discussion

miR-140 is located in the intronic region of the gene Wwp2 which codes for a ubiquitin E3 ligase (Inui et al., 2018). It is highly expressed in skeletal and chondrocyte cells, and it is crucial for bone development (Nakamura et al., 2011). Previous studies have suggested that stem cells originating from adipose tissues can differentiate into either osteoblasts or adipocytes (Uccelli et al., 2008; Keating, 2012). MicroRNAs (miRNAs) have been found to perform essential regulatory functions in adipocyte development (Tang et al., 2009; Lee et al., 2011). Furthermore, other studies have found that gga-miR-140-5p promotes intramuscular adipocyte differentiation via targeting retinoid X receptor gamma (Zhang et al., 2018) and miR-140-5p may be involved in the adipogenic and osteogenic lineage differentiation of human adipose-derived stem cells (Li et al., 2017b). These studies indicate that miR-140 may function in the differentiation process of adipocytes. However, little is known regarding miR-140’s accurate role in adipogenesis. In this study, our data illustrated that miR-140-5p level was induced in 3T3-L1 cells during adipocyte differentiation and that miR-140-5p was necessary for 3T3-L1 preadipocytes to sustain their adipogenic differentiation and lipogenesis. Our data illustrated that miR-140-5p might be involved in adipogenesis. An earlier study also suggests that this microRNA is upregulated during adipogenesis in hASCs (Li et al., 2017b). However, it remains unknown how miR-140-5p promotes adipocyte differentiation and whether it has any role in adipogenesis.

Our investigation of the impact of miR-140-5p on adipocyte differentiation revealed its significance in the determination of cell destiny. Over-expression of miR-140-5p in preadipocytes induced lipogenesis and adipocyte differentiation, but the knockdown of endogenous miR-140-5p impeded lipogenesis and adipocyte differentiation. Our data illustrated that miR-140-5p was a positive modulator for lipogenesis and adipocyte differentiation. Previous research has reported that Wnt/β-catenin, mTOR signaling pathways, PPARγ, and C/EBPs are the signaling pathways affected by miRNAs (Wang et al., 2013; Li et al., 2015; Liang et al., 2015; Gu et al., 2016). Consistent with this, our data illustrated that miR-140-5p elevated the expression of adipogenic transcription factors such as PPARγ and C/EBPs. Here, we demonstrated that miR-140-5p promotes adipocyte differentiation by directly targeting and regulating PDGFRα, a critical functional marker of adipocyte progenitor cells (Berry and Rodeheffer, 2013; Sun et al., 2017). Based on the Targetscan data resource analysis, we screened the miR-140-5p target genes which have complementary sites of miR-140-5p in the 3′UTR region. Through luciferase assays, and qRT-PCR along with Western blot analyses, we confirmed that PDGFRα was a miR-140 target gene regulating adipocyte differentiation.

Some studies have reported that PDGFRα activation inhibits adipogenesis, thus promoting the generation of profibrotic cells (Iwayama et al., 2015; Hogarth et al., 2019). However, another research study has shown that PDGFRα promotes adipocyte progenitor cell differentiation to generate beige fat (Gao et al., 2018). In this study, PDGFRα expression was reduced under the induction of miR-140-5p during adipocyte development, thus increasing the adipogenesis-related genes (C/EBPs and PPAR) expression as well as enhancing lipid aggregation in 3T3-L1 preadipocytes. Furthermore, during adipocyte differentiation, over-expression of PDGFRα reversed the miR-140-5p–induced increase in adipogenesis-related gene expression. Our findings suggest that miR-140-5p can limit the suppression of adipogenesis-related gene expression and intracellular lipid accumulation by PDGFRα, thus contributing to adipogenesis. Consistent with this idea, PDGFRα is involved in the differentiation of cardiomyocyte differentiation (Xu et al., 2019). However, the signaling pathway in response to PDGFRα activation is still largely unknown, and thus the molecular processes regulating the expression and function of adipogenesis-related genes remain to be further investigated in future research.

In conclusion, our data illustrates that miR-140-5p is involved in adipocyte differentiation and the development of adipocyte precursor cells into adipocytes, thus promoting lipogenesis. Here, we identified PDGFRα as an miR-140-5p direct target involved in adipocyte differentiation. Therefore, miR-140-5p might be a new potential target for the clinical diagnosis and treatment of obesity and related metabolic diseases. Since our results are limited to *in vitro* experiments, future studies will explore its role in obesity-related metabolic diseases. Nonetheless, our findings elucidate an essential function of miR-140-5p in adipocyte differentiation and adipogenesis. Our results indicate that inhibition of miR-140-5p expression via an aptamer might be a potential therapeutic strategy to treat obesity and obesity-related metabolic diseases.

## Materials and Methods

### Cell Culture, Cell Transient Transfection, and Adipocyte Differentiation

All cells were cultured in high-glucose DMEM (Life Technologies, Carlsbad, United States) containing streptomycin, penicillin, and 10% FBS in a 5% CO_2_ humid incubator at 37°C. Cells were inoculated into dishes or plates, and then transfected with vectors, siRNA, or mimics on day 2 post inoculation. We used lipofectamine RNAiMAX and lipofectamine 2000 for cell transient transfection (Invitrogen, Carlsbad, United States). Two days after the 3T3-L1 cells attained confluence, cell differentiation was induced by an activation cocktail consisting of 100 nM insulin, 1 μM Dex, 0.5 mM IBMX (Sigma-Aldrich, Germany), and 10% FBS. The medium was renewed with 10% FBS DMEM enriched with 100 nM insulin every 2 days until the cells grew into mature adipocytes.

### RNA Extraction and qRT-PCR

The RNAiso Plus reagent (Takara, Japan) was adopted to isolate low–molecular-weight RNA along with total RNA. The cDNA was prepared using the Prime Script RT Reagent Kit with gDNA Eraser (Takara, Japan). The qRT-PCR was conducted with SYBR Green qPCR Mix reagent (Monad, Wuhan). The comparative-Ct approach (2^-ΔΔCt^ approach) was employed to compute gene relative expression levels.

### Western Blots

Whole-cell protein was isolated from 3T3-L1 cells by using the lysis buffer (Beyotime, China). We determined the protein concentrations with a BCA protein assay kit (Beyotime, China). SDS-PAGE was performed to separate protein lysates, and the obtained protein was then transferred to the NC or PVDF membrane (Millipore, United States). Then, the membrane was inoculated for 2 hours with 5% skimmed milk, and then inoculated overnight with anti-C/EBPα (18311-1-AP, Proteintech, Chicago), anti-PDGFRα (bsm-52829R, Bioss, China), anti-C/EBPδ (36,790, Signalway Antibody), or anti-C/EBPβ (23431-1-AP, Proteintech, Chicago) at 4°C. After three washes, the membrane was inoculated with 1:5,000 or 1:10,000 dilution of the secondary antibody for 1.5 h at RT (room temperature). After three washes again, the membrane added with enhanced chemiluminescence (Bio-Rad, United States) was exposed in the imaging system.

### Luciferase Reporter Assay

The mouse PDGFRα 3′UTR was added to the psiCheck2 vector to construct the luciferase report vector (Promega). The potential docking site seed sequences of miR-140-5p were found to be 5′-AACCACT-3'. After the 48-h transfection, the activity of luciferases was assessed in the Dual Luciferase Enzyme Reporter Assay System (Promega).

### Identification of miR-140-5p Target Genes

miR-140-5p potential downstream target genes were identified based on data from the following three databases: PicTar (https://pictar.mdc-berlin.de/), MicroRNA.org (https://www.mirbase.org/index.shtml), and TargetScan (http://www.targetscan.org/vert_72/).

### Oil Red O Staining Assay

The cells were rinsed with PBS buffer and fixed in 10% formalin for 1 h at 4°C after cell differentiation. After fixation, the cells were rinsed thrice with PBS and stained with 0.35 percent oil red O (Sigma-Aldrich, Germany) for 1 hour at RT. The cells were assessed and photographed after washing with distilled water.

### Statistical Methods

All the data were given as the mean ± standard deviation (SD) of at least three replicates. All the data were analyzed and plotted using GraphPad Prism 6. Student’s two-tailed *t*-test was performed to assess the statistically remarkable differences between groups. *p < 0.05, **p < 0.01, and ***p < 0.001 represented the three levels of significant differences.

## Data Availability

The original contributions presented in the study are included in the article/Supplementary Material; further inquiries can be directed to the corresponding author.
