# Supplementary material for: microRNA-140 Regulates PDGFRα and Is Involved in Adipocyte Differentiation
Source: Front Mol Biosci. 2022 Jun 27;9:907148. doi: 10.3389/fmolb.2022.907148 (PMC9271708; doi:10.3389/fmolb.2022.907148)
Supplement: Supplementary file 1 [file DataSheet1.docx]

Supplementary Materials

**Supplementary Figures**


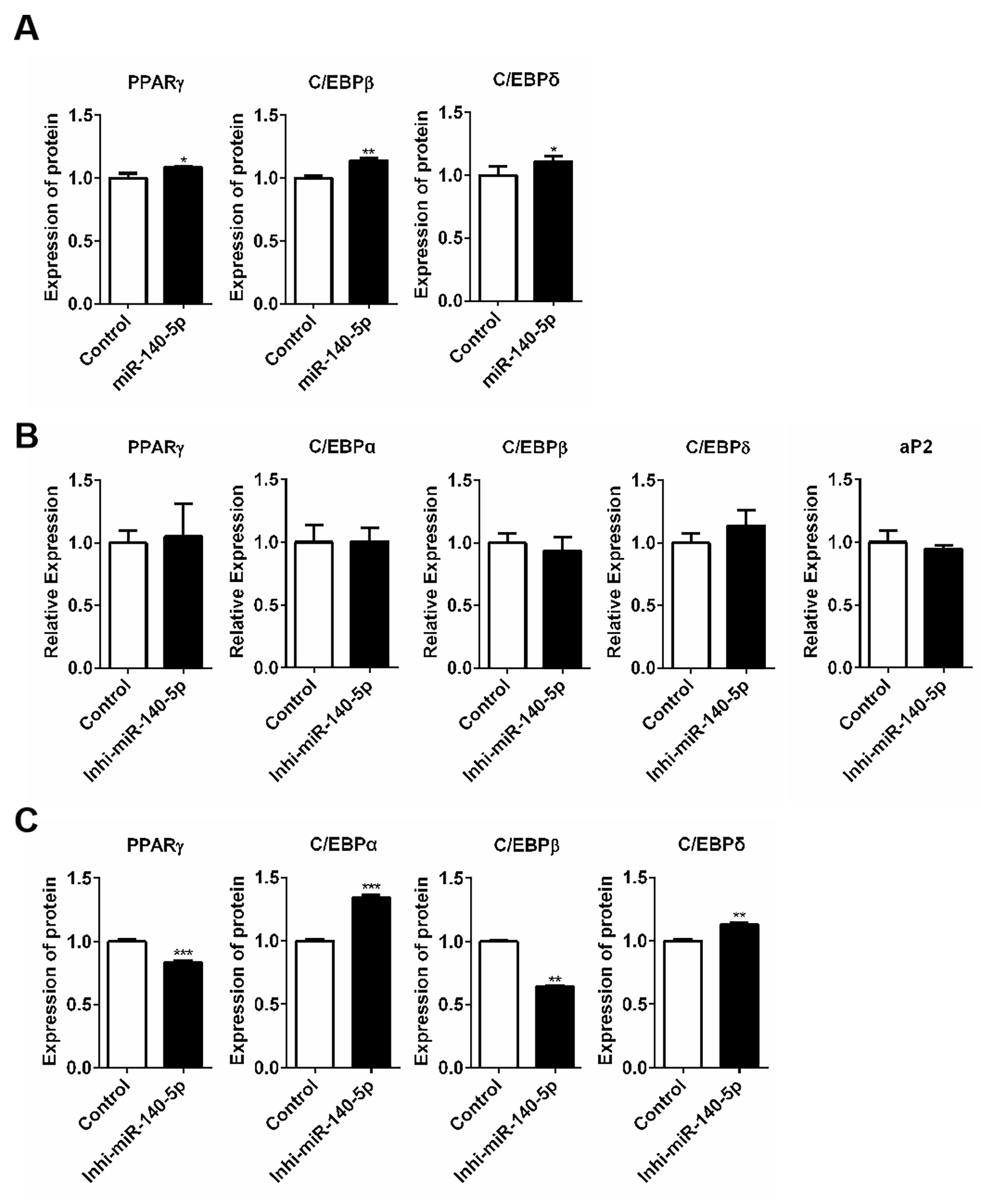


**Figure S1 miR-140-5p is a transcriptional modulator of adipogenesis**

1. The relative protein expression of different adipocyte differentiation markers was measured by western blotting after transient transfection with miR-140-5p mimics or control into 3T3-L1 cells.
2. Realtime PCR analysis of adipogenic genes expression in 3T3-L1 cells transduced with miR-140-5p inhibitors (Inhi-miR-140-5p) or negative control mimics (Control).
3. The relative protein expression of different adipocyte differentiation markers was measured by western blotting after transient transfection with miR-140-5p inhibitors (Inhi-miR-140-5p) or control into 3T3-L1 cells.

Data are expressed as means ± SD, n = 3 independent experiments containing three replicates. Significant difference is presented at the level of *p < 0.05, **p < 0.01 and ***p < 0.001 by two-tailed Student’s t test.


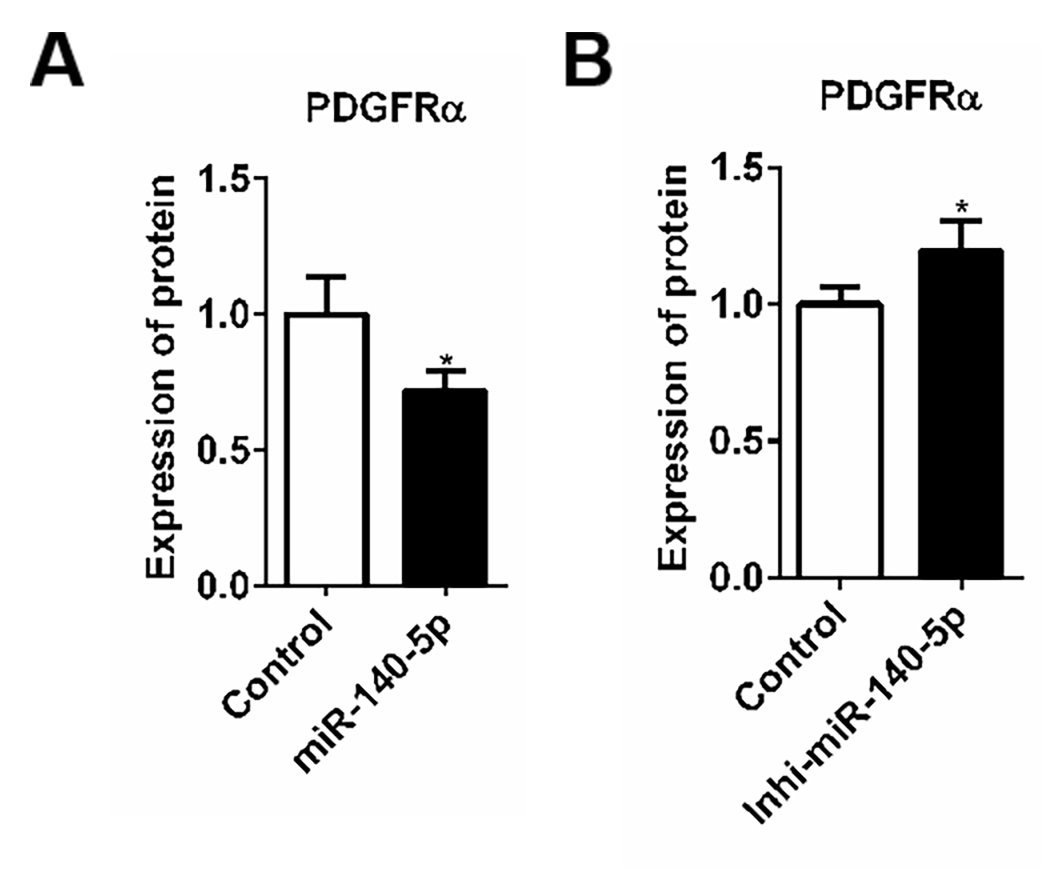


**Figure S2 miR-140-5p Directly Targets** **PDGFRα**

1. The relative protein expression of PDGFRα was measured by western blotting after transient transfection with miR-140-5p mimics or control into 3T3-L1 cells.
2. The relative protein expression of PDGFRα was measured by western blotting after transient transfection with miR-140-5p inhibitors (Inhi-miR-140-5p) or control into 3T3-L1 cells.

Data are expressed as means ± SD, n = 3 independent experiments containing three replicates. Significant difference is presented at the level of *p < 0.05 by two-tailed Student’s t test.


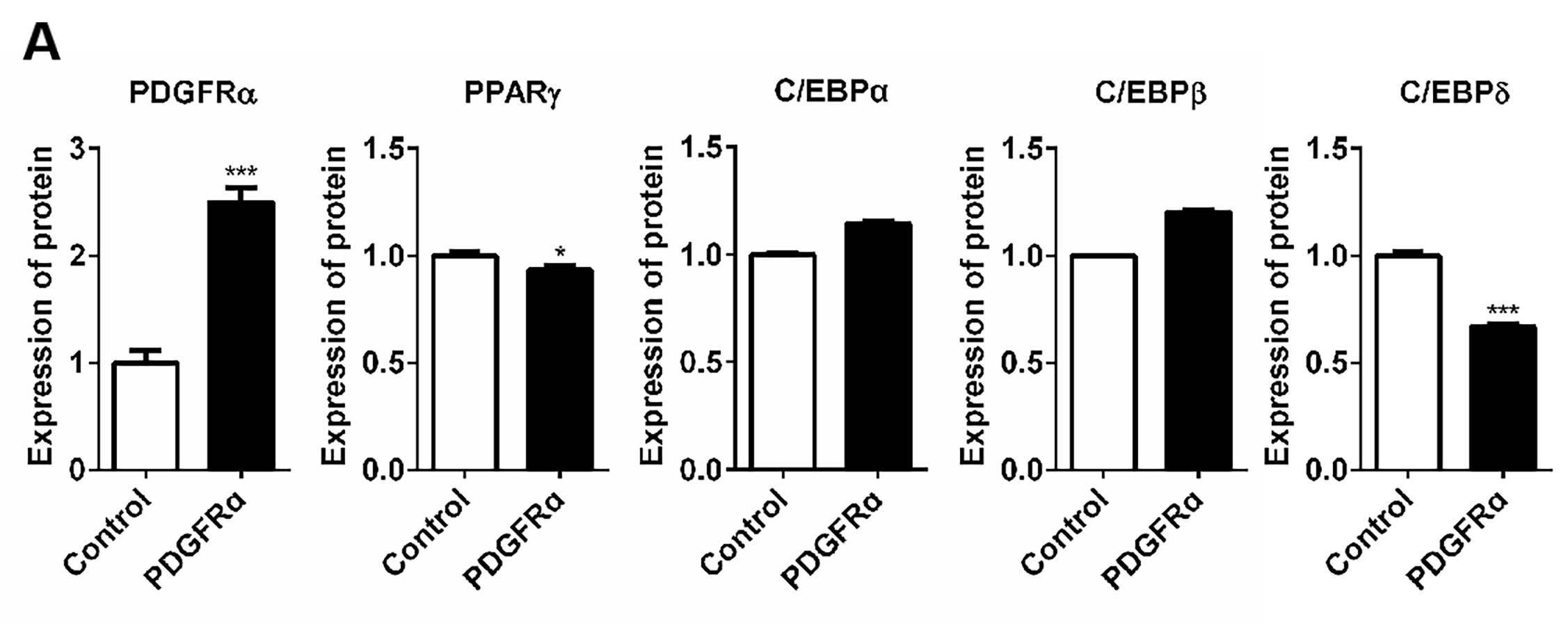


**Figure S3 Effect of PDGFRα on adipogenic related genes expression and lipid droplet synthesis in 3T3-L1 Cells.** The relative protein expression of different adipocyte differentiation markers was measured by western blotting after transient transfection with pEGFP-N1-PDGFRα (PDGFRα) or control into 3T3-L1 cells.

Data are expressed as means ± SD, n = 3 independent experiments containing three replicates. Significant difference is presented at the level of *p < 0.05 and ***p < 0.001 by two-tailed Student’s t test.


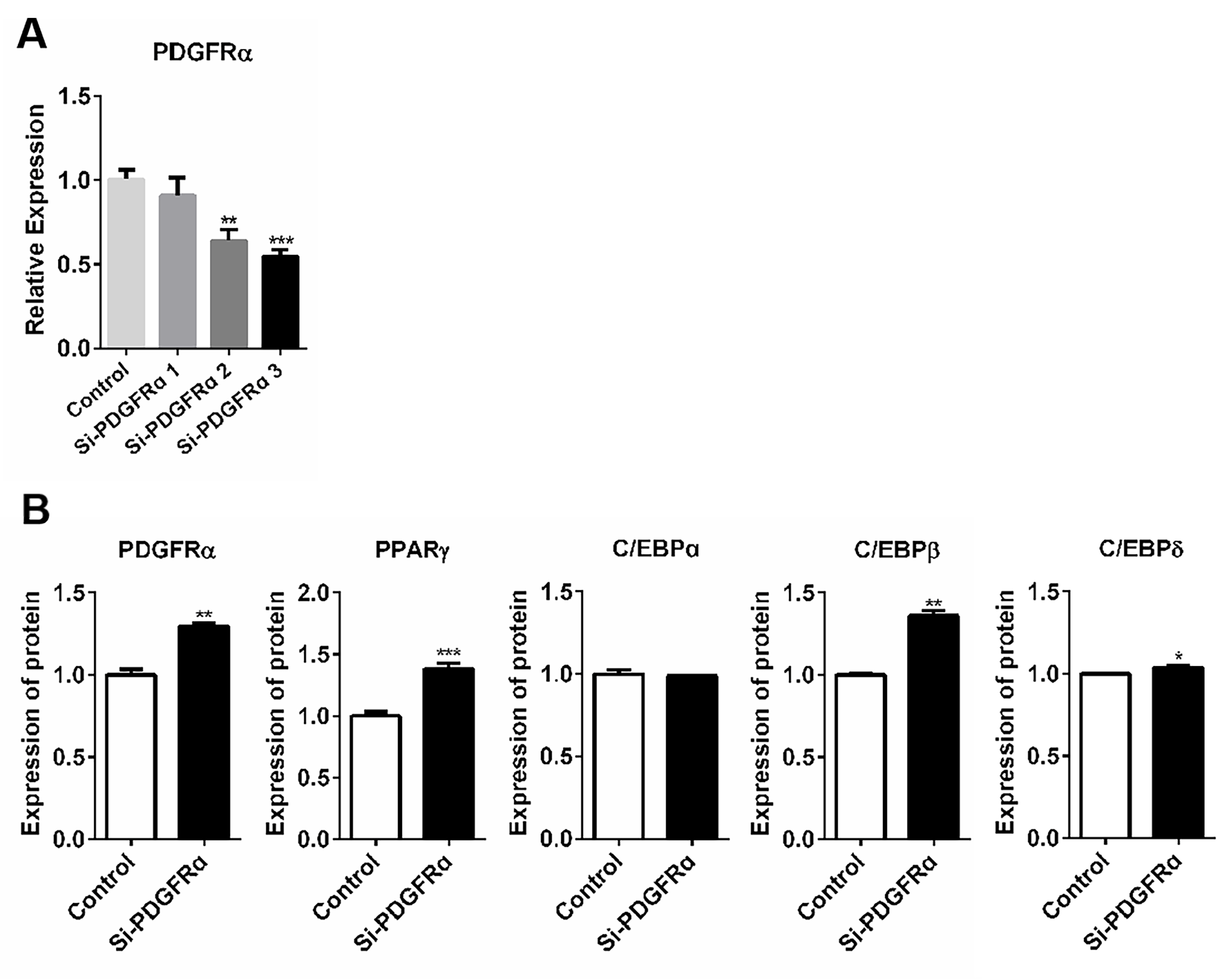


**Figure S4 PDGFRα depletion increase adipocyte differentiation markers expression and lipid droplet synthesis**

1. The relative protein expression of PDGFRα was measured by western blotting after transient transfection with PDGFRα siRNA 1 (Si-PDGFRα1), PDGFRα siRNA 2 (Si-PDGFRα2), PDGFRα siRNA 3 (Si-PDGFRα3) and control into 3T3-L1 cells.
2. The relative protein expression of adipocyte differentiation markers in 3T3-L1 cells transfected with PDGFRα siRNA (Si-PDGFRα) or control.

Data are expressed as means ± SD, n = 3 independent experiments containing three replicates. Significant difference is presented at the level of *p < 0.05, **p < 0.01 and ***p < 0.001 by two-tailed Student’s t test.
